# Perceiving numerosity does not cause automatic shifts of spatial attention

**DOI:** 10.1007/s00221-021-06185-7

**Published:** 2021-08-05

**Authors:** Michele Pellegrino, Mario Pinto, Fabio Marson, Stefano Lasaponara, Fabrizio Doricchi

**Affiliations:** 1 Department of Psychology, “Sapienza” University of Rome, Via dei Marsi, 78, 00185 Rome, Italy; 2 Neuroimaging Laboratory, Department of Physiology and Pharmacology, Sapienza” University of Rome, Rome, Italy; 3 Research Institute for Neuroscience, Education and Didactics, Fondazione Patrizio Paoletti, Assisi, Italy; 4 Libera Università Maria Santissima Assunta—LUMSA, Roma, Italy; 5 Laboratorio di Neuropsicologia della Attenzione, Fondazione Santa Lucia IRCCS, Via Ardeatina 306, 00179 Rome, Italy

**Keywords:** Numerosity, Space-Number Association, Attention, Number magnitude

## Abstract

It is debated whether the representation of numbers is endowed with a directional-spatial component so that perceiving small-magnitude numbers triggers leftward shifts of attention and perceiving large-magnitude numbers rightward shifts. Contrary to initial findings, recent investigations have demonstrated that centrally presented small-magnitude and large-magnitude Arabic numbers do not cause leftward and rightward shifts of attention, respectively. Here we verified whether perceiving small or large non-symbolic numerosities (i.e., clouds of dots) drives attention to the left or the right side of space, respectively. In experiment 1, participants were presented with central small (1, 2) vs large-numerosity (8, 9) clouds of dots followed by an imperative target in the left or right side of space. In experiment 2, a central cloud of dots (i.e., five dots) was followed by the simultaneous presentation of two identical dot-clouds, one on the left and one on the right side of space. Lateral clouds were both lower (1, 2) or higher in numerosity (8, 9) than the central cloud. After a variable delay, one of the two lateral clouds turned red and participants had to signal the colour change through a unimanual response. We found that (a) in Experiment 1, the small vs large numerosity of the central cloud of dots did not speed up the detection of left vs right targets, respectively, (b) in Experiment 2, the detection of colour change was not faster in the left side of space when lateral clouds were smaller in numerosity than the central reference and in the right side when clouds were larger in numerosity. These findings show that perceiving non-symbolic numerosity does not cause automatic shifts of spatial attention and suggests no inherent association between the representation of numerosity and that of directional space.

## Introduction

A central issue in current studies on mathematical cognition is whether the mental representation of numbers is endowed with an inherent spatial component so that, for example, in left-to-right reading cultures smaller numbers are automatically positioned to the left of larger ones on a mental number line (MNL). The automatic generation and use of MNLs was initially suggested by the introspective reports that Sir Francis Galton (Galton 1880a, b) collected in healthy adults who experienced vivid forms of MNL upon just hearing or reading a number. From an experimental standpoint, the functional association between space and numbers, i.e., the Space-Number Association (SNA), is best and most reliably highlighted in the Spatial Numerical Association Response Code effect (SNARC, Dehaene et al. 1993). The SNARC shows that when judging the magnitude or parity of numbers in a given number range, healthy humans are faster at providing manual responses on the left side of space when a number is small in magnitude and faster at responding on the right side when a number is large in magnitude. The original interpretation of the SNARC (Dehaene et al. 1993) was that it arises from the correspondence, or non-correspondence, between the left/right position that numbers would inherently occupy along the MNL and the left/right spatial position of the motor response that is associated with the magnitude or parity of numerical targets.

Over the years, this interpretation has been supplemented and qualified by other proposals (for review of this issue, see Kadosh et al. 2008; Wood et al. 2008; Fattorini et al. 2016; Pinto et al. 2019a, b, 2021a). These have emphasised the role played by specific factors in the genesis of the SNARC, like the influence of culturally acquired conceptual polarities that would include congruent concepts like “small” and “left” rather than incongruent ones like “small” and “right” (Proctor and Cho 2006; Gevers et al. 2010) and the fact that the SNARC arises at the response-selection level (Keus et al. 2005; Gevers et al. 2006). Nonetheless, these improved interpretations of the SNARC all assume, in line with the original interpretation, that the processing of the magnitude or the parity of a number inherently and automatically activates a corresponding spatial representation of the same number, so that in western cultures, smaller numbers would be automatically mentally placed to the left of larger ones (for a recent computational model sharing the same assumption, see Chen and Verguts, 2010).

Around 17 years ago, the “inherent spatial positioning” assumption of number magnitudes was reinforced by the finding that the mere perception of centrally presented small-magnitude Arabic numbers would produce automatic shifts of attention towards the left side of space and that of large numbers towards the right side (the Attentional-SNARC effect, Fischer et al. 2003). Nonetheless, ensuing investigations have suggested that, when observed, the Attentional-SNARC effect is not automatic and is rather strongly influenced by top-down strategic factors (Ristic et al. 2006; Galfano et al. 2006), so that it can be reversed just by asking participants to imagine a reversed MNL with small numbers to the right of larger ones. Most importantly, a series of more recent studies did not replicate this effect. Basing on data from the original study by Fischer et al. (2003), van Dijck and colleagues (2014) estimated a priori the effect size and the precise number of participants (31) needed to obtain an optimal statistical power (*β* = 0.90). Forty-three participants were examined, and no Attentional-SNARC effect was found. In two experiments with the original paradigm, also Zanolie and Pecher (2014) found no Attentional-SNARC. In our lab, in three different studies, we did not replicate the Attentional-SNARC (Fattorini et al. 2015, 2016; Pinto et al. 2018). In a re-analysis of the 174 participants tested in our lab (Pellegrino et al. 2019), we also failed to detect any influence of factors like finger counting style, imagery vividness, and learning style on the direction or consistency of the Attentional-SNARC. Bayesian analyses confirmed these negative findings. Similarly, in a registered replication report (Colling et al. 2020) that grouped data collected in 17 different laboratories from all over the world, no evidence of the Attentional-SNARC effects was found in a total sample of 1105 participants. Taken together, these studies convincingly point out that the mere perception of Arabic numbers does not evoke SNAs.

It has been suggested that the exact notion of quantity that is conveyed by symbolic Arabic numbers is rooted in a phylogenetic older non-symbolic system that allows grasping the approximate “numerosity” of perceptual items in the environment (Nieder 2005), both when these are presented simultaneously and when they are sequentially distributed in time (Nieder et al. 2006; Binetti et al. 2012). Though approximate, the non-symbolic “numerosity” sense is highly adaptive because it allows deciding rapidly, for example, whether a location in the environment contains more or less food items than another one. In the present study, we wished to investigate whether non-symbolic numerical magnitudes, represented by small-numerosity or large-numerosity arrays of dots presented at central fixation, can automatically trigger lateral shifts of spatial attention in a way that is consonant to initial findings with symbolic Arabic numbers (Fischer et al. 2003). In the first experiment, we used a conventional Attentional-SNARC task with central non-symbolic numerosity cues rather than symbolic Arabic ones (i.e., Dots Attentional-SNARC task). The second experiment (i.e., dots colour task) consisted of an adaptation to the study of lateral shifts of attention in humans of the task that Rugani et al. (2015) devised to investigate the influence of non-symbolic numerosities on the spatial orientation of chicks. In that study, chicks were initially adapted to search food behind a central panel depicting a specific numerosity reference (e.g., five dots). Following adaptation, two lateral panels were presented. In different trials, both panels depicted numerosities that were lower or higher than the initial central numerosity reference. The presence of significant explorative biases for the left-side or the right-side panel was investigated. The authors observed a leftward bias when panels depicted numerosities lower than the adapted central numerosity reference and a rightward bias when panels depicted numerosities higher than the reference. Although these findings have now not been replicated in pigeons and blue-jaybirds (Lazareva et al. 2020) and monkeys (Beran et al. 2019), here through an ad hoc modification of the task by Rugani et al. (2015), in which we considered as dependent variable RTs in humans rather than turning motor responses in animals, in Experiment 2 we re-tested whether perceiving low vs high numerosities causes automatic leftward and rightward shifts of spatial attention, respectively.

## Experiment 1

### Materials and methods

#### Participants within

We calculated the total number of participants that would have been necessary to run comparisons among the factors that were considered in the study. This analysis showed that a total of 23 participants would be needed to have a power of 0.95 for detecting an effect size *f* = 0.40 when employing the traditional 0.05 criterion of statistical significance for repeated measures within factors ANOVA. We tested 28 healthy right-handed students (19 females, 9 males, mean age = 22.3 years, SD = 1, 60 years) from the University “Sapienza” in Rome who participated in the study. They all had normal or corrected to normal vision and were naive to the experimental hypothesis.

#### Procedure and stimuli

Participants performed a “Dots Attentional-SNARC task” that included non-symbolic numerosity central cues in the form of clouds of dots. Dot clouds were generated using a modified version of the script for MATLAB developed by Gebuis and Reynvoet (2011). Stimuli were presented on a 15-in. colour 6546 IBM monitor using MATLAB software and the Cogent toolbox to control the presentation of stimuli and record manual responses. Participants performed the task in a sound-attenuated room with dim illumination and positioned their head on a chin rest at a viewing distance of 57.7 cm from the screen.

**Fig. 1:**
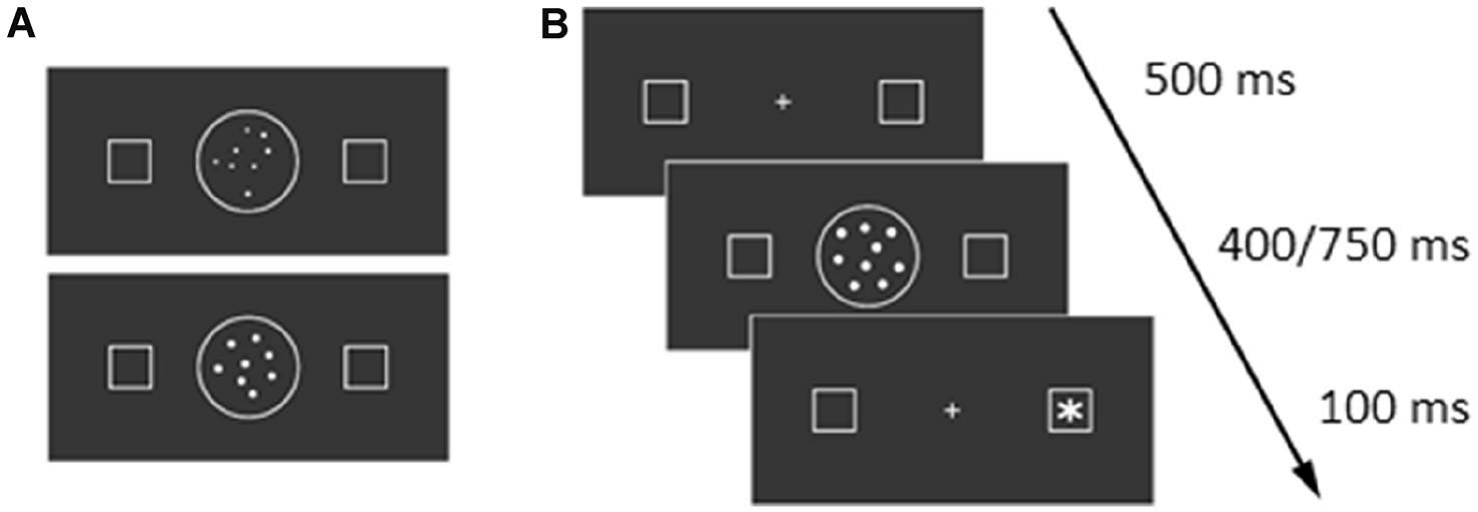
**A** Examples of variable (up) and fixed (down) dots condition. **B** Example of trial

The Dots Attentional-SNARC task was composed of two blocks, one for each of two different dots condition. In the first condition (henceforth “Fixed” condition), dots had all the same dimension (diameter = 0.2°). In the second condition (henceforth “Variable” condition), dots had a diameter that varied between 0.1° and 0.25°. In this second condition, the visual area occupied by dots was the same across the different numerosities included in the task. All dots were presented in a circular area with a diameter of 2.85° (Fig. 1A). The order of experimental conditions was counterbalanced between participants. Each block consisted of 128 experimental trials (32 for each numerosity) and 32 catch trials (20% of total trials).

At the beginning of each trial, a central fixation cross (0.4° × 0.4°) and two lateral square boxes (1° × 1°) were presented for 500 ms. The centre of each box was positioned 5° to the left and the right of the central fixation cross. At the end of this 500 ms period, one of four numerosities (i.e., 1, 2, 8 or 9) replaced the central fixation cross for 500 ms. After the numerosity cue, the central fixation cross was shown again, and after a variable Cue–Target Interval (CTI 400 or 750 ms), a white asterisk–target (0.5° × 0.5°) was presented inside one of the lateral boxes for 100 ms (Fig. 1B). Participants were asked to press the space bar with their right index finger as quickly as possible in response to the target. Before testing, as in Fischer et al. (2003), participants were instructed that numerosities presented at fixation did not predict the target locations and were irrelevant to the detection task. A training session was administered before the experiment.

## Results

Catch trials (false alarms), trials in which no response was made (misses) and trials in which RTs were above or below two standard deviations from the individual mean of RTs were considered outliers and were excluded from the analysis.  ~ 4% of trials were discarded from the analysis.

In a first step, individual mean RTs were entered in a dots condition (variable, fixed) × CTI (400, 750) × target side (left, right) × numerosity (1, 2, 8, 9) repeated measures ANOVA. All post hoc analyses were performed using the Bonferroni method.

**Fig. 2:**
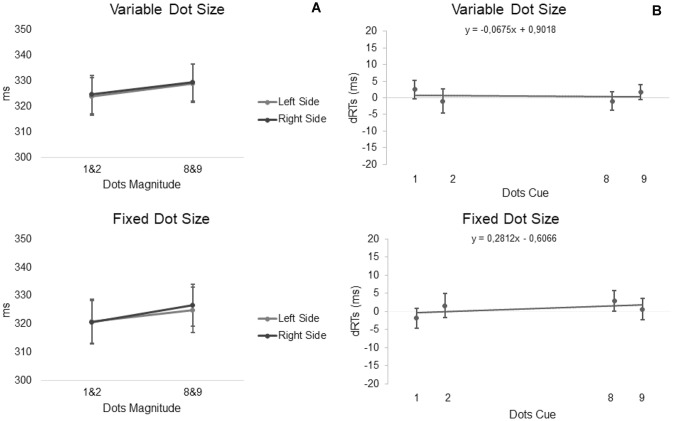
**A** Average RTs (with SE) to targets in the left and right side of space as a function of the magnitude of central numerosities-cues, i.e., small (1, 2) and large (8, 9), for Variable Dot Size in the upper panel and Fixed Dot Size in the lower panel. **B** Slope describing the difference between RTs to targets in the right side of space minus targets in the left side of space (dRT in ms), plotted as a function of the number of central numerosities-cues, for Variable dot size in the upper panel and fixed dot size in the lower panel

The main effect of CTI [*F* (1, 27) = 24.66, *p* < 0.001, *η*_*p*_^2^ = 0.48] was significant, showing that manual RTs were faster with 750 CTI as compared to 400. Also the main effect of numerosity [*F* (3, 81) = 6.24, *p* < 0.001, *η*_*p*_^2^ = 0.19] was significant pointing out that manual RTs were faster for panel with one dot as compared to eight dots (post hoc 1 320.09 ms vs. 8 326.93 ms, *p* < 0.05) and nine dots (post hoc 1 320.09 ms vs. 9 327.96 ms, *p* < 0.001). No main effect of Dots Condition was found [*F* (1, 27) = 0.52, *p* > 0.05, *η*_*p*_^2^ = 0.02]. In a second step, we investigated the presence of the Dots Attentional-SNARC effect by entering individual mean RTs in a numerosity (smaller, larger) × target side (left, right) × CTI (400, 750 ms) repeated measures ANOVA. In this case, no significant main effect or interaction was found (all *p* > 0.05, mean RTs averaged between CTI smaller-left side = 323.76 ms, smaller-right side = 324.54 ms, larger-left side = 328.88 ms, larger-right side = 329.24 ms) (Fig. 2).

In addition, the Dots Attentional-SNARC effect was also evaluated using a regression analysis (Lorch and Myers 1990): in this analysis, a significant negative slope (Fias 1996; Ito and Hatta 2004) indexes a significant Attentional-SNARC effect. We considered individual differential RTs (dRTs) obtained by subtracting the average RTs recorded in trials with left-side targets from average RTs recorded in trials with right-side targets and calculated individual linear regression slopes using numerosity as the predictor variable and dRTs as the criterion variable (Fig. 2B). A one-sample *t* test showed that both the slopes calculated in variable and in the fixed dots conditions (mean = −0.06, SD = 2.0, mean = 0.28, SD = 2.5) did not show the Dots Attentional-SNARC effect [*t* (27) = −0.18, *p* > 0.05, *t* (27) = 0.59, *p* > 0.05].

We also performed Bayesian hypothesis testing using JASP (JASP Team 2019) (version 0.11.1). Individual mean RTs were entered in a dots condition (variable, fixed) × numerosity (smaller, larger) × target side (left, right) Bayesian repeated measures ANOVA. This analysis confirmed that the likelihood of the alternative hypotheses (a) numerosity × target side interaction or a dots condition × numerosity × target side interaction) were less likely (BF_10_ = 0.023 and BF_10_ = 0.31, respectively) than the null hypothesis.

## Experiment 2

### Materials and methods

#### Participants

We calculated the total number of participants that would have been necessary to run comparisons among the considered factors. This a priori analysis showed that a total of 23 participants would be needed to have a power of 0.95 for detecting an effect size *f* = 0.40 when employing the traditional 0.05 criterion of statistical significance for repeated measures within factors ANOVA. We tested 28 healthy right-handed students (24 females, 4 males; mean age = 22 years, SD = 1.89 years) from the University “Sapienza” in Rome who participated in the study. They all had normal or corrected to normal vision and were naive to the experimental hypothesis.

#### Procedure and stimuli

Experiment 2 consisted of a “Dots Colour task”. Dots conditions were the same as Experiment 1, though, in Experiment 2 the diameter of dots in the variable condition varied between 0.1° and 0.3°. In this experiment, we used three numerosities (2, 5 and 8): two of them (2 and 8) were presented in the lateral panels (see below), while the third (5) was always presented as a reference in the central panel. Four different rotations were adopted for each numerosity (0°, 90°, 180°, 270°) to minimize the possibility that a fixed and specific spatial distribution of stimuli acted as a directional cue.

**Fig. 3:**
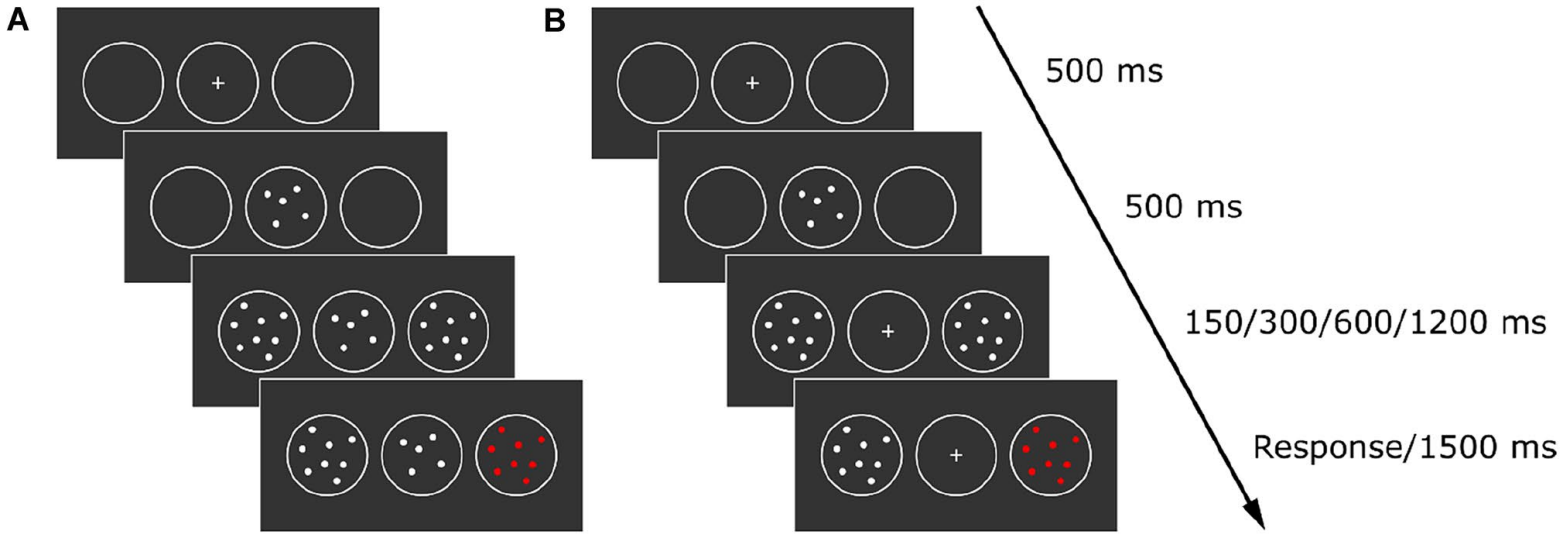
**A** Example of trial in 5With task condition. **B** Example of trial in 5Without task condition

The “Dots Colour task” consisted of two experimental conditions, named “5With”" and “5Without”, administered both for “Fixed” and “Variable” dots conditions that were intermixed within each block. The task was composed of four blocks, and each block consisted of 128 experimental trials (64 for each numerosity) and 32 catch trials (20% of total trials). The order of experimental conditions was counterbalanced between participants. At the beginning of each trial, a central circle-box (with a central 0.4° × 0.4° fixation cross at its centre) and two lateral circle-boxes were presented for 1 s. The diameter of all boxes was 2.85°. The centre of each lateral box was positioned at 4° from the centre of the central box. At the end of this 1 s period, a cloud with five dots replaced the central fixation cross for 500 ms. At this point, in the “5With” condition, the central dot clouds remained on the screen together with the lateral clouds. After a variable CTI (150, 300, 600 or 1200 ms), one of the lateral clouds went red (Fig. 3). In the “5Without” condition, the central dot cloud disappeared simultaneously with the appearance of the two lateral small-numerosity (two dots) or large-numerosity (eight dots) dot clouds in the two lateral boxes.

Participants were asked to press the space bar with their right index finger as quickly as possible in response to the target (the change of colour, a maximum time of 1500 was allowed for response). Before testing, participants were instructed that numerosities presented at fixation did not predict the target locations and were irrelevant to the detection task. A training session was administered before the experiment.

## Results

 ~ 1% of trials were discarded from the analysis due to catch trials, misses or outliers. Individual mean RTs were entered in a Task Condition (5With, 5Without) × dots condition (variable, fixed) × cti (150, 300, 600, 1200 ms) × numerosity (2, 8) × target side (left, right) repeated measures ANOVA. All post hoc analyses were performed using the Bonferroni method.

The main effect of Dots Condition [*F* (1, 27) = 10.68, *p* < 0.05, *η*_*p*_^2^ = 0.28] was significant, showing that manual RTs were faster in the fixed as compared to the variable condition. Also, the main effect of CTI [*F* (3, 81) = 81.97, *p* < 0.001, *η*_*p*_^2^ = 0.75] was significant, pointing out that RTs were faster with 300 ms CTI (all *p* < 0.05) as compared to all the other CTI, and slower with the 600 ms CTI (all *p* < 0.05) as compared to all the other CTIs. Finally, the main effect of numerosity [*F* (1, 27) = 17.42, *p* < 0.001, *η*_*p*_^2^ = 0.39] was significant, highlighting that RTs were faster with 8 dots-panels as compared to the two dots-panels.

The task condition (5With, 5Without) × dots condition (variable, fixed) interaction was significant [*F* (1, 27) = 7.49, *p* < 0.05, *η*_*p*_^2^ = 0.22], showing that variable dots in 5With condition led to slower RTs as compared to all the other conditions (post hoc: 5With-variable 395.21 ms vs. 5With-fixed 388.99 ms, 5With-variable 395.21 ms vs. 5Without-variable 388.97 ms, 5With-variable 395.21 ms vs. 5Without-fixed 388.09 ms, all *p* < 0.001). More in detail, within the 5With condition, fixed dots led to faster RTs than variable dots (post hoc: fixed: 388.99 ms, vs variable: 395.21 ms, *p* < 0.001). At variance, no difference between variable and fixed dots RTs (*p* > 0.05) was found in the 5Without task condition.

Also, the dots condition × numerosity interaction was significant [*F* (1, 27) = 43.19, *p* < 0.001, *η*_*p*_^2^ = 0.62], showing that eight dots-panels in fixed condition led to faster RTs as compared to all the other conditions [post hoc: fixed-8 381.51 ms vs. fixed-2: 395.57 ms, fixed-8 381.51 ms vs. variable-2 391.39 ms, fixed-8 381.51 ms vs. variable-8 392.78, all *p* < 0.001]. In detail, within the Fixed condition, eight dots-panels led to faster RTs as compared to two dots-panels (post hoc fixed-8 381.51 ms vs. fixed-2 395.57 ms, *p* < 0.001). At variance, no difference between eight dots-panels and two dots-panels RTs was found in the variable condition (post hoc: variable-8 392.78 vs. variable-2 391.39 ms, *p* > 0.05).

**Fig. 4:**
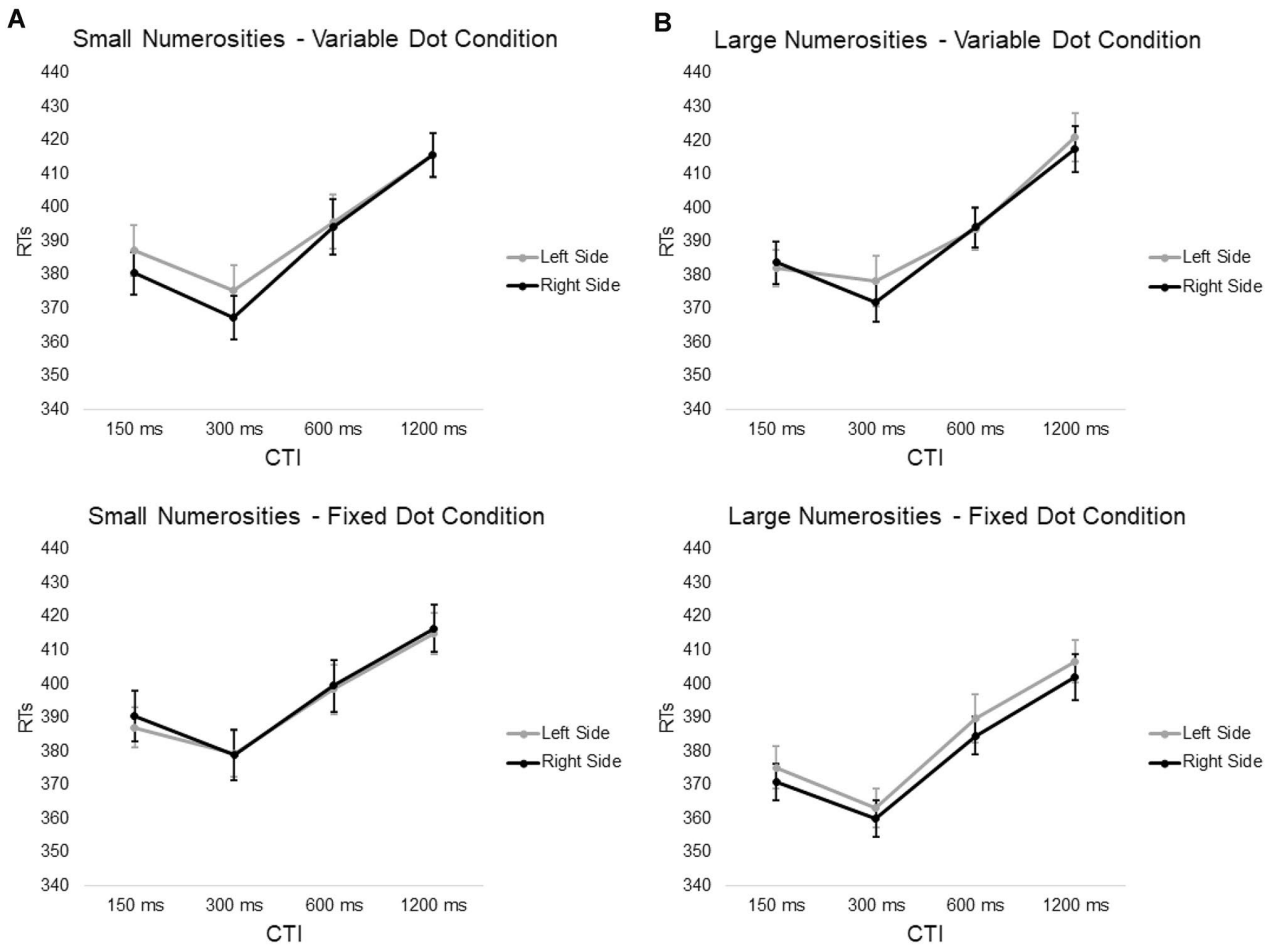
Average RTs (with SE) to targets presented in the left and right side of space plotted as a function of the magnitude of central numerosity cues, **A** Low (1, 2) or **B** High (8, 9), and Cue-target interval for variable dot condition (first row) and fixed dot condition (second row)

**Fig. 5:**
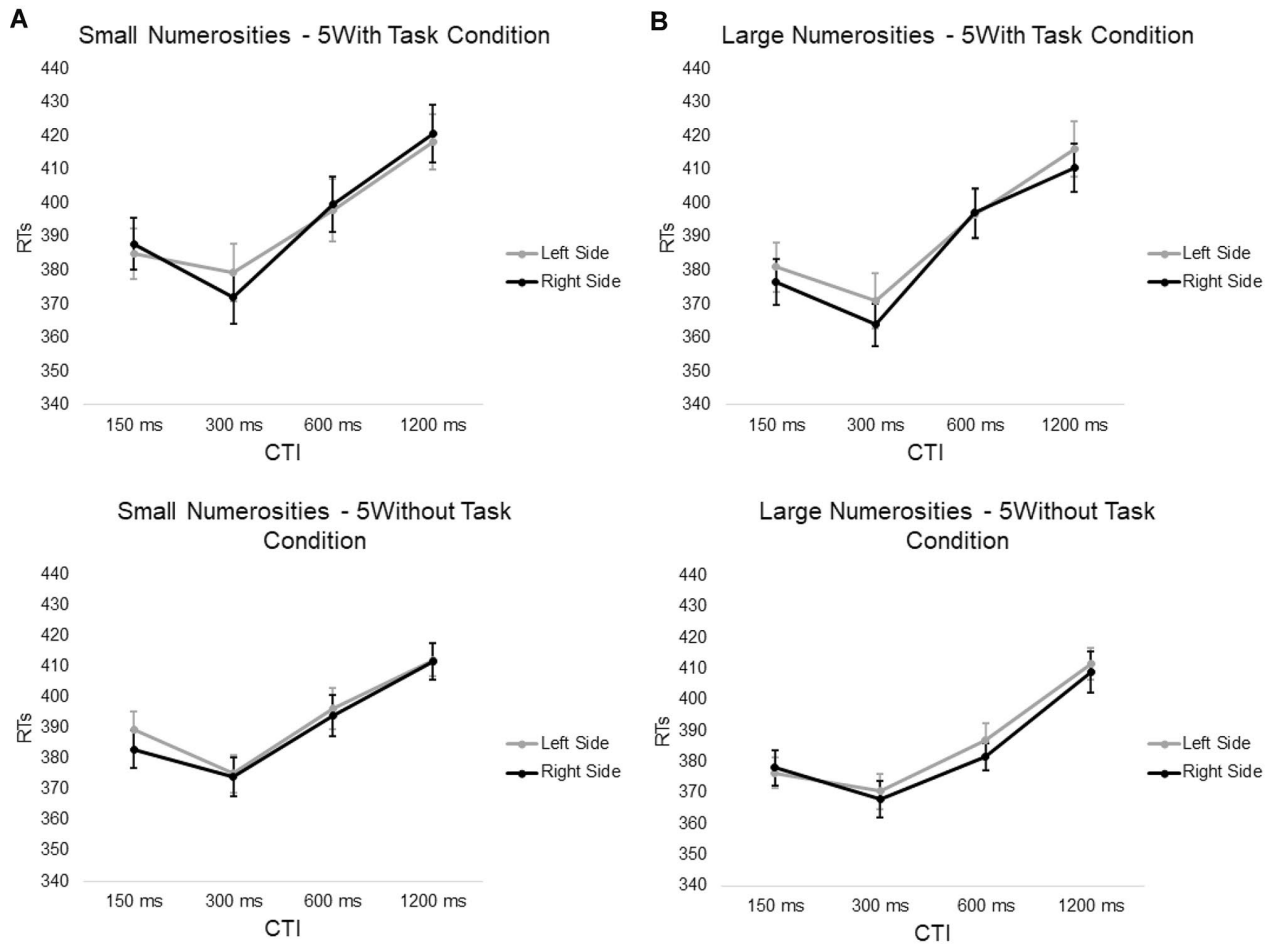
Average RTs (with SE) to targets presented in the left and right side of space plotted as a function of the magnitude of central digit cues, **A** Low (1, 2) or **B** High (8, 9), and Cue-target Interval for “5With” task condition (first row) and “5without” task condition (second row)

The numerosity × target side interaction, the dots condition × numerosity × target side interaction (Fig. 4) and the task condition × numerosity × target side interaction were all not significant (all *p* > 0.12, mean RTs averaged between dots and task condition: smaller-left side = 394.66 ms, smaller-right side = 392.52 ms, larger-left side = 388.81 ms, larger-right side = 387.19 ms) (Fig. 5).

We also performed Bayesian hypothesis testing using JASP (JASP Team 2019) (version 0.11.1). Individual mean RTs were entered in a task condition (5With, 5Without) × dots condition (variable, fixed) × numerosity (smaller, larger) × target side (left, right) Bayesian repeated measures ANOVA. This analysis highlighted that the interactions of interest were all less likely than the null hypothesis in explaining our data (task condition × dots condition × numerosity × target side, BF_10_ = 0.47, dots condition × numerosity × target side, BF_10_ = 0.87, task condition × numerosity × target side, BF_10_ = 0.45, numerosity × target Side, BF_10_ = 0.17).

## Discussion

The results of the two experiments reported in the present study show that perceiving non-symbolic numerosities in the shape of small-numerosity or large-numerosity clouds of dots presented at central fixation does not trigger directionally congruent lateral shifts of spatial attention: small numerosities do not speed up the detection of attentional targets in the left side of space, and large numerosities do not speed up the detection of targets in the right side. In Experiment 2, using a modification of the paradigm devised by Rugani et al. (2015) for the study of chickens, we also found that presenting a central numerosity reference embedded within two lateral numerosities, so to create a horizontal sequence of numerosities, does not generate directionally congruent inspective biases. When both lateral dot-clouds are less numerous than the central reference, participants are not faster at detecting colour changes in the left-side cloud, and when lateral dot-clouds are more numerous than the central reference, participants are not faster at detecting changes in the right-side cloud. Besides, the permanence or disappearance of the central reference during the presentation of lateral clouds did not affect these results. In both experiments, panels with large numerosity were associated with faster RTs than panels with small ones. Similarly, panels including dots with equal size were associated with faster RTs than panels including dots variable in size. Both effects can be easily explained by considering that both large-numerosity panels and panels with dots of equal and fixed-size conveyed a greater amount of visual stimulation.

If one considers that in everyday life, the use of symbolic Arabic numbers and their left-to-right arrangement in word strings is certainly more common and diffused than an equivalent use of non-symbolic numerosities, the negative result of the present study with a non-symbolic Attentional-SNARC task should not be considered that astonishing, also in the light of extensive negative findings that have now been collected in Attentional-SNARC experiments with symbolic numbers (van Dijck et al. 2014; Zanolie and Pecher 2014; Fattorini et al. 2015, 2016; Pinto et al. 2018; Pellegrino et al. 2019; Colling et al. 2020). In line with this observation, it is important to note that while a reliable SNARC effect is almost invariably reported with symbolic Arabic numbers, much less coherent findings are reported with non-symbolic numerosities notwithstanding, at variance with the Attentional-SNARC task, the SNARC task requires the explicit the use of contrasting left/right spatial codes for response selection. In an initial report using non-symbolic numerosities, i.e. arrays of geometrical shapes, Mitchell and co-workers (2012) found a significant SNARC effect when participants had to judge the upward/downward orientation of shapes through a left vs right response selection choice. In contrast, no effect was found when the colour of shapes had to be discriminated (both in the orientation and colour judgement task, numerosity of stimuli was task-irrelevant). Nonetheless, in a more recent investigation, the same research group (Cleland et al. 2020) failed to replicate the SNARC effect in the orientation task and concluded that non-symbolic representations of number do not offer a direct and automatic route to numerical–spatial associations. In another study, Zhou et al. (2016) asked participants to judge, through a left vs right spatial response choice,  whether two dots clouds (i.e., 11, 14, 17, 20, 23, 26, and 29 dots), that were sequentially presented at central fixation, were “matched” or “non matched” in numerosity. The results were not homogenous as the authors only found faster right-hand responses to large non-symbolic magnitudes, whereas no difference was found for small non-symbolic magnitudes. Nemeh et al. (2018) asked healthy adult participants to judge, through a left vs right spatial response selection, whether a target-cloud of dots (i.e., 15, 20, 25, 36, 45 and 60 dots) contained “less” or “more” dots than a previously presented reference-cloud (i.e., 30 dots). These authors found faster responses with the left hand to targets (i.e., 15, 20, or 25 dots) smaller than the reference and faster responses with the right hand to targets larger (i.e., 36, 45 and 60) than the reference, thus highlighting a significant SNARC effect for non-symbolic magnitudes. Taken together, these results are far from being coherent and point out that even in a task like the SNARC where the use of contrasting spatial left vs right response codes is explicitly required, non-symbolic numerosities fail to activate a spatial representation of number magnitudes reliably.

Here it is also important to consider the few studies that have reported evidence favouring the idea that non-symbolic numerosities provoke lateral shifts of attention. Using non-symbolic low-numerosity (two dots) and large-numerosity (nine dots) central cues, Bulf and co-workers (2014, 2016) reported the presence of the Attentional-SNARC both in healthy adults and in 8–9-month-old infants. In these studies, the time interval between the occurrence of the lateral target and the landing of ocular fixation on the target was used as a dependent variable. Nonetheless, it is important to note that the infra-red monitoring of eye position was run at a very low time resolution with a sampling rate of 120 Hz which corresponds to controlling eye-position on each 8.3 ms (please note that manual RTs are taken at 1 ms resolution). Besides, an eye movement was considered to land on the lateral target when it actually landed on an area of 12.6° around the target, which was only 3° in size and was presented in a circular box of 6° in diameter. Finally, it is important to note that using the landing time of saccades as a measure of shifts of spatial attention, rather than the latency of saccadic onset, might not be fully adequate and reliable because the time of saccadic landing also includes the duration of the eye movement which spuriously adds to the attentional-ocular index that is provided by saccadic latency. Due to these important caveats, Bulf and co-workers’ studies (2014, 2016) are difficult to be interpreted and should be replicated using a high time resolution recording of saccadic latencies and considering only saccades that actually land on lateral targets.

In a more recent study, Di Giorgio et al. (2019) adapted the paradigm devised by Rugani and colleagues (2015) for the study of chickens, to the study of newborns. They habituated newborns (51 h old, on average) to view two identical lateral patterns made of 12 black dots. Dots were included in a white square (size 30.3° × 30.3°), and the left and right square were separated by 13.65°. Once habituated, children were presented with couples of identical squares that included 4 or 36 dots. Di Giorgio et al. (2019) reported that following habituation, newborns showed an ocular preference for the left square when both squares included four dots (i.e., fewer dots than the reference) and the right square when both squares included 36 dots (i.e., more dots than the reference). According to the authors, these findings were in line with those previously reported by Rugani and co-workers (2015) in chickens and would point out that in humans, the left-to-right organised MNL originates from “pre-linguistic and biological precursor in the brain”.

In a study on 7-month infants, de Hevia et al. (2014) presented visual arrays of coloured rectangular-shaped items that varied in numerosity from 4 to 16. Numerosity stimuli were presented sequentially from left to right (Experiment 1) or from right to left (Experiment 2). Sequences were organised so that there was a sequential increase or decrease in numerosity. The authors highlighted that infants showed a preference (i.e., longer average looking time) only for increasing numerical sequences presented from left to right side. In contrast, no corresponding preference was present for decreasing sequences going from right-to-left. Because of this incomplete matching between magnitude and directional sequencing, the authors concluded that this preference seems to be “malleable” and can be modified or cancelled by cultural and task factors “*in line with the idea that the early bias to link numerical order to spatial directionality is plastic and easily modifiable by experiential and cultural factors*”. In an ensuing study, de Hevia et al. (2017), run one main experiment (Exp. 1) followed by four control experiments. Data from 16 newborns (taken from an initial sample of 80) were included in the final analyses. In Exp. 1, eight newborns were initially adapted to the central presentation of a short horizontal line (total duration 60 s) that was matched with the repeated presentation of short sound (duration = 1.4 s), while the other eight newborns were adapted to the central presentation of a long horizontal line that was matched with a long sound (duration 4.3 s). At the end of adaptation, newborns who were exposed to short lines were presented with two test trials, one with a long line in the left side of space and one with a long line in the right side of space. Vice versa, newborns who were adapted with long lines were presented with short lines during the two test trials. Adapting and test stimuli were presented at a distance of 60 cm. Based on the combination of visual on-line and off-line scoring of looking times, the authors concluded that newborns show a looking preference for the left-side test when previously adapted to long lines and of the right test when adapted to short lines.

Though potentially appealing, it is important to note that the above-summarised results with newborns and infants were obtained through direct online and/or offline (i.e. video recording) visual scoring of new-borns ocular exploration and await independent replications with more precise and reliable infra-red recordings of ocular fixations (for a review on this methodology, see Gredebäck et al. 2009). This technique might also allow collecting data from a larger number of trials, thus enhancing the validity and reliability of measures. In addition, future studies should carefully consider that in newborns: a) the ideal viewing distance to see an object or a face is between 20 and 25 cm (Haynes et al. 1965; Dobson and Teller 1978; this criterion is respected in the study by Di Giorgio et al. 2019); (b) the correct perception of bilateral stimuli, like those used by Di Giorgio et al. (2019) is based on effective mechanisms of binocular rivalry that in young infants reach full development only at three months of age (Fox et al. 1980, Yang et al. 2016).

Contrary to the findings that seem to point at a phylogenetical and ontogenetical preference in placing small magnitudes on the left side of space and large magnitudes on the right side, several recent studies have highlighted no systematic directional preference. For example, using a computerized version of the task devised by Rugani et al. (2015), Beran and colleagues (2019) failed to find directional left to right space-number preferences in rhesus macaques and capuchin monkeys. In addition, Lazareva and colleagues (2020) found robust evidence for a flexible, rather than fixed, left-to-rightspatial representation of magnitudes with considerable individual variability in domestic pigeons (*Columba livia*) and blue jays (*Cyanocitta cristata*). Similarly, Gazes and co-workers (2017) trained apes (orangutans and gorillas) to discriminate the larger or the lower between two numerosities, one presented on the left and one on the right side of a touch screen. These authors found marked inter-individual variability in the preferential association of specific numerosities with a specific side of space. Some apes were faster at touching/selecting smaller numerosities on the left side and larger numerosities on the right side of space. In contrast, other apes showed an opposite pattern of preference, so that no left-to-right or right-to-left preference was observed in the whole group.

Taken together, the negative results of these recent animal studies suggest that discrepant results between our adaptation to the study of attention in humans (Experiment 2) of the task proposed by Rugani et al. (2015) and the results reported by these authors in chicks cannot merely or solely explained by differences in the dependent variables considered in the two studies, i.e. RTs in humans vs. turning motor responses in chicks, or by other factors such as the absence of reward release or the tachistoscopic presentation of stimuli in the human task.

Recent studies in healthy humans have provided support to the idea that rather than being native, the directional left-to-right or right-to-left organization of MNLs is triggered by the use of left/right codes in the numerical task at hand (Fattorini et al. 2015, 2016; Pinto et al. 2018, 2019b). These studies show that reliable horizontal MNLs are evoked only when left/right spatial codes are contrasted to select one out of two spatially defined and competing motor responses, like in the SNARC task (Fattorini et al. 2015, 2016; Pinto et al. 2018, 2019b), or to release or uphold a non-spatially defined response like in Go/No-Go task (Fischer and Shaki 2017; Shaki and Fischer 2018; Pinto et al. 2019a, 2021a). In line with these conclusions, it is worth noting that healthy humans display similar lateral biases in the bisection of horizontal visual lines (Longo and Laurenco 2007) and in the mental bisection of number intervals, when these intervals are defined by placing their endpoints one to the left and one to the right side of a visual horizontal line: nonetheless this correlation entirely disappears when the endpoints of number intervals are defined verbally, i.e. with no visual-spatial cues suggesting their left-to-right arrangement (Rotondaro et al. 2015). The result of a recent study (Pinto et al. 2021a, b) in right brain-damaged patients with left spatial neglect (Bartolomeo et al. 2007; Doricchi et al. 2008; Lecce et al. 2015; Silvetti et al. 2016) reinforces the idea that the use of left/right spatial response codes is crucial in triggering the SNA. This study shows that in a Magnitude Comparison SNARC that requires the use of contrasting left/right spatial codes for the selection of one out of two lateral motor responses, patients with neglect are significantly slower at deciding that the number “4” is smaller than the number “5” than deciding that the number “6” is larger than the number “5”. This result replicates previous findings and suggests that in neglect patients responses to number "4" are slowed down because this number is mentally placed to the left of the central mental numerical reference (i.e., “5”). Nonetheless, the same patients show no “4” vs “6” RTs asymmetry when the same Magnitude Comparison task is performed in a Go/No-Go procedure that requires pressing or upholding a central response button without requiring the left/right spatial response codes. This finding suggests that the directional left-to-right representation of number magnitudes is evoked by the very use of left/right spatial codes and that the SNARC effect does not derive from a primary congruency or incongruence between the independent spatial representation of numbers and spatial-response codes, but that it is rather secondary to the transfer of spatial response codes to the representation of number magnitudes that, otherwise, would not be endowed with an inherent spatial component (Aiello et al. 2012, 2013; Fattorini et al. 2015; Pinto et al. 2018, 2019a, b, 2021a, b).

In conclusion, the result of the present study adds to growing evidence suggesting that the mental spatial representation of numerical magnitudes is not “native” but rather generated by cultural and context-dependent factors such as reading habits and task requests. Here we note that assuming a phylogenetical and ontogenetically native left-to-right organisation of the MNL should necessarily imply that individuals belonging to right-to-left reading cultures and who organise their MNL from right-to-left must undergo a radical developmental re-organization of their native left-to-right MNL. In addition, since in natural settings, small and large numerosities are never preferentially located to the left, and the right of an agent viewpoint, respectively, flexible rather than fixed directional associations between numbers and space seem far more adaptive.

## Data Availability

Data will be available upon request to the authors.

## Code availability

Code will be available upon request to the authors.
